# Art Therapy for Women Who Have Experienced a Spontaneous Miscarriage: A Thematic Analysis of Reflections

**DOI:** 10.3390/bs16050801

**Published:** 2026-05-18

**Authors:** Monika Tekutienė, Daiva Jakavonytė-Staškuvienė

**Affiliations:** 1 Faculty of Medicine, Utena University of Applied Sciences, 28142 Utena, Lithuania; monikatekutiene@gmail.com; 2 Faculty of Nursing, Lithuanian University of Health Sciences, 44307 Kaunas, Lithuania; 3 Education Academy, Vytautas Magnus University, 44248 Kaunas, Lithuania

**Keywords:** art therapy, spontaneous pregnancy loss, miscarriage, reflection

## Abstract

Spontaneous pregnancy loss is the most common pregnancy complication; 15–25% of clinically confirmed pregnancies end in miscarriage, and when early, undetected pregnancy losses are included, the miscarriage rate can reach as high as 30–60% of all pregnancies. Women who lose their babies in the early stages of pregnancy often experience this as a bereavement. However, in Lithuania, 95.2% of women who had experienced a miscarriage and participated in the study did not receive any psychological support; only 4% of those surveyed stated that they did not need it. However, both in Lithuania and abroad, there is still a lack of research on women’s emotional state following a spontaneous pregnancy loss and the impact of interventions on it. Research question: What experiences emerge in the thematic analysis of women who have experienced a spontaneous miscarriage? A qualitative study was conducted using the inductive thematic analysis method. The study participants underwent a 10-session group art therapy programme. After each art therapy session, the study participants reflected on their experiences related to their miscarriage by analyzing the drawings they had created. Verbal data from the reflections were recorded, then transcribed and analysed according to identified themes. The research participants were four women who had experienced a spontaneous pregnancy loss. The study analyses the reflections of three women who participated in all sessions. The thematic analysis revealed four themes characterising the women’s core experiences of spontaneous pregnancy loss: defensiveness, the grieving process, a complicated relationship with oneself and others, and awareness and finding meaning.

## 1. Introduction

Women who have experienced a miscarriage feel stigmatized, ashamed, and guilty, and avoid talking about this painful experience all over the world (Gaižauskaitė, 2019; Bilardia et al., 2021; Seftel, 2020). That many women, regardless of their culture, education or upbringing, are reluctant to talk about their loss. For this reason, research on this topic can contribute to revealing the experiences of women who have experienced a spontaneous pregnancy loss.

One in five pregnancies ends in miscarriage. For women, this can be a profound and painful loss, experienced as the death of a long-awaited and planned child. Consequently, women feel a sense of injustice, wonder why this has happened to them specifically, and blame themselves for perceived mistakes that may have affected the baby’s development. Studies have noted a correlation between spontaneous pregnancy loss and various psychological disorders, as well as an increased risk of developing mental health conditions (Bright et al., 2020; Coenen, 2018; Kouriatis & Brown, 2013; Kulathilaka et al., 2016; Seftel, 2020). In 2010, a study was conducted to investigate the psychological state of 280 participants immediately after a miscarriage, and at 3 months, 6 months and 12 months later. It was found that women experience severe distress following a miscarriage: 55% of women immediately after the termination of pregnancy, 25% after 3 months, 17.8% after 6 months, and 10.8% after 1 year (Lok et al., 2010). Another review of 29 studies (Shetty et al., 2025), involving 35,375 participants, found that within six weeks of a miscarriage, 32.5% of women experienced anxiety, 30.1% experienced depression, and 33.6% experienced stress. It was noted that the prevalence of these mental health disorders was higher in low- and middle-income countries.

However, women who have experienced a spontaneous termination of pregnancy face not only psychological difficulties but also social problems (Gaižauskaitė, 2019; Bilardia et al., 2021; Seftel, 2020). If the first pregnancy ends in this way, a woman not only feels grief and the associated emotions but also faces the thought that she may never have children again due to supposed infertility (Kübler-Ross et al., 1972; Matulaitė, 2013). In this case, one of the most difficult things is the overwhelming uncertainty, the question of whether she will be able to conceive next time, or whether the next pregnancy will end in the same way. The belief that one is infertile brings about major changes in a woman’s life and triggers low self-esteem, feelings of worthlessness, and symptoms of depression (Bright et al., 2020; Coenen, 2018; Kouriatis & Brown, 2013; Kulathilaka et al., 2016; Seftel, 2020; Taylor et al., 2020). Due to their reduced self-esteem, women may wish to isolate themselves, as being around friends who have successfully had children can often be too painful. Consequently, they withdraw from society, unconsciously limiting their social circle.

Another significant problem is that, when faced with a non-progressive pregnancy, patients often undergo dilation and curettage, they are given medication to induce uterine cleansing, or are left to wait for spontaneous cleansing; in this way, the physical problem is resolved, but women leave healthcare facilities without knowing what to do about their emotional distress. Gaižauskaitė (2019), who studied women’s emotional state following a miscarriage, states that 95.2% of women who experienced a miscarriage in Lithuania received no psychological support. Four out of 99 women (4%) who did not receive support stated that they did not need such support. A survey published in Australia, which examined the availability of support for 399 women who had experienced a spontaneous termination of pregnancy, revealed two main findings: (1) more than half of the women (59%) were not offered any information about support or peer support organisations at the time of their miscarriage, nor were they given a referral or the opportunity to access the services of counsellors or psychologists; (2) more than half (57%) did not receive follow-up care or emotional support, unless they were asked how they were feeling (Bilardia et al., 2021). Another issue is recurrent miscarriages. One study (Tavoli et al., 2018) involved 105 women who had experienced recurrent miscarriages and 105 women who had never had a miscarriage. The social and demographic characteristics of both groups were similar. Women who had experienced recurrent miscarriages reported significantly higher levels of psychological stress. Women who had experienced recurrent miscarriages reported a significantly lower quality of life in many areas: physical condition, general health, vitality, social functioning, emotional state and mental health (Tavoli et al., 2018).

Art therapy is a practice carried out by an art therapist to promote health, integrating knowledge, skills and practices from the fields of art, medicine, psychology and psychotherapy, with creative activity as its primary means of expression and communication. A study conducted in 2024 examined whether art therapy can improve the quality of life for women who have recently experienced a pregnancy loss. The study involved 60 women, divided into an intervention group and a control group. Over eight weeks, the intervention group participated in four structured art therapy sessions, whilst the control group received only standard care. The results show that women who participated in art therapy experienced a significant improvement in quality of life across biopsychosocial domains (Zahmatkesh et al., 2024). Furthermore, it is important to emphasise that the benefits of art therapy for women experiencing symptoms of depression have already been established (Blomdahl et al., 2018), and it has been found that this form of therapy reduces fear of childbirth (Wahlbeck et al., 2020). It is important to note that women who have experienced a miscarriage feel a multitude of overwhelming emotions that are very difficult to put into words, so they do not talk about it. Undoubtedly, for them to open up, a safe environment and a trusting relationship are required. Art therapy provides a safe space, a relationship, and the means to express emotions (Hogan, 2020). When analysing the artwork created by the client together, there is a sense that it is not the person themselves who is being discussed, but the artwork and its symbols; consequently, the person can relax and express their thoughts more freely. In this way, what had been repressed in the subconscious becomes clearer, easier to name and recognise. It is also important to emphasise that the art therapy methodology helps to transform the current situation: to provoke more positive emotions, boost self-confidence, and create new ways of solving problems. In 2018, researchers investigated the effects of phenomenological art therapy on individuals diagnosed with severe and moderate depression (Blomdahl et al., 2018). It emerged that for this group of patients, such activities helped to re-establish a connection with themselves; through reflection on their drawings, they were able to engage in an inner dialogue, specifically distinguishing between what is known and real, and what is merely unrealistic speculation or assumption. Such a dialogue with oneself provides a greater sense of control over one’s own life, helps to accept reality and, at the same time, offers the opportunity to change one’s perspective on it and discover alternative ways of solving problems. When it comes to those experiencing grief, it is important to note that talking alone is not enough to overcome it (Coenen, 2018). Researchers have recognised that artistic practices not only improve mood but are also highly effective in restoring a person’s overall well-being (Zahmatkesh et al., 2024). Creative engagement is described as a way of discovering and utilising each person’s natural inner healing resources, thereby returning to a state of wholeness; creative self-expression is considered one of the most effective means of releasing the body and processing emotions accumulated during the grieving process (Hogan, 2020). Qualitative analysis has revealed three key roles of the creative process in therapy: a space for the client’s grieving process; a channel of communication that influences the art therapist’s experience and the therapeutic relationship; and a shared space in which the client and therapist create a new narrative (Bat Or & Garti, 2018).

On the other hand, a person’s connection with themselves and their self-perception depend greatly on their relationships with other people. However, the loss of connection with the developing life within the womb is associated not only with the loss of their relationship but also with a threat to the mother’s own connection with herself (Hogan, 2020; Seftel, 2020; Zahmatkesh et al., 2024). Women who have experienced a miscarriage feel a sense of loss of self; they may no longer feel a connection with their own bodies, so visual and kinesthetic stimulation—such as seeing different colours, sticking, drawing, and touching different textures—helps them to feel their own bodies. Creativity is also a way to fill an inner void, isolation and loneliness. To illustrate this point, we can refer to a study (Arnold, 2023). Its aim was to investigate how professional visual artists experience loss and how this experience influences their creative work. The study involved eight professional artists who had experienced the death of a loved one. The study revealed that artistic creation can become an important form of grief processing, helping to express complex emotions, better understand and make sense of the loss, establish a new relationship with the deceased, and share one’s experience with others (Arnold, 2023). Furthermore, women who have experienced a pregnancy loss face societal pressure to erase the loss from their memory, on the grounds that they never even saw their baby (Seftel, 2020). For this reason, the opportunity for a bond to form between mother and child—regardless of whether it is referred to as an embryo or a foetus—is lost. Through visual creation, women can establish this bond by creating a tangible object that serves as a reminder of it. In this way, the loss is given meaning, and the baby finds a place in the woman’s life (Seftel, 2020). It is important to mention the study conducted by Hogan (2020), whose participants were women who had experienced a spontaneous miscarriage. They attended art therapy sessions daily for two weeks. Ultimately, it was found that the participants’ self-confidence and motivation had increased, and they felt less social isolation. As their well-being was assessed using the Warwick–Edinburgh Mental Well-being Scale both before and after the study, a 37% increase was observed (Hogan, 2020). Thus, it can be argued that guided art therapy sessions can be an effective intervention, promoting healing, the restoration of a connection with oneself and one’s environment, and the finding of meaning in loss among women who have experienced a pregnancy loss.

## 2. Theoretical Framework of the Study

**Concepts of the phenomenological and social constructivist paradigms on which the study is based.** This work is grounded in the phenomenological paradigm. This approach focuses on revealing the meaning of lived experience. Phenomenology analyzes the experience of returning to a specific stage of life and helps to reestablish a direct connection with reality (Geniušas, 2021; Moran, 2000). Phenomenology enables the unveiling of the essences inherent in the phenomena of consciousness. A phenomenon is defined as everything we perceive or experience, regardless of whether it is real or not (Mickūnas & Jonkus, 2014). The phenomenological approach seeks to describe how a particular phenomenon manifests itself in human consciousness and what meanings the person experiencing it attributes to it; therefore, subjective experience is paramount in the study (A. Giorgi et al., 2017; A. P. Giorgi & Giorgi, 2003). Within this paradigm, the researcher seeks to describe the participants’ experiences as accurately as possible by applying phenomenological reduction—temporarily setting aside prior theoretical assumptions and preconceptions in order to analyse the units of meaning revealed in the descriptions provided by the participants (A. Giorgi et al., 2017; A. P. Giorgi & Giorgi, 2003). For this reason, our work first involved conducting the research, followed by a detailed review of the academic literature. In summary, it can be stated that this paradigm is particularly suitable for investigating sensitive and deeply personal experiences, such as pregnancy loss, as it allows for a more detailed understanding of how women reflect on their experiences and what aspects these encompass. For this reason, based on this paradigm, verbal data can be analysed to understand subjective reality (Geniušas, 2021; Mickūnas & Jonkus, 2014; Moran, 2000).

In phenomenological research, great importance is placed on the researcher’s own interest in the topic; however, the topic must not be too sensitive (Matulaitė, 2013). In order to examine the experiences of women who have experienced miscarriage in a deep, multifaceted, and comprehensive manner, it is necessary to remove the “glasses of prior knowledge,” “set aside” one’s own experience, put “expertise” to one’s side, and allow oneself to be guided by the research participant (Matulaitė, 2013). One of the authors of this article has experienced a miscarriage, so before beginning to research this topic, it was important to answer the question of whether she was prepared to take on the role of researcher. Since the author herself had undergone psychotherapy following her own loss, and later art therapy as well. Furthermore, five years had passed since the event, and she already had two children, so she clearly understood that researching this phenomenon would not resolve her personal issues. While conducting the research, it was important to constantly “check in” and reflect on her personal relationship with the phenomenon. For this reason, she discussed it regularly during supervision sessions. This allowed her to constantly monitor whether the researcher was identifying with the stories told by the research participants, and how the emotional state of not only the participants but also the researcher changed after art therapy sessions. Thus, constant self-reflection and supervision made it clear that there is a distinct boundary between the researcher’s life and that of the other participants, and that she is able to maintain a professional distance from the participants’ stories.

**The relationship between art therapy and a study of the experiences of women who have experienced spontaneous miscarriage.** The “stages of grief” (Kübler-Ross et al., 1972) is a model comprising five stages: denial, anger, bargaining, depression, and acceptance. However, this model is viewed critically today because grief is not a linear process. According to the theory of meaning reconstruction, grief is not merely an emotional response to loss, but an active process during which a person creates a new relationship with the loss. This involves the effort to make sense of the experience and integrate it into one’s identity (Neimeyer et al., 2006). Grief is not a structured, linear process, and every loss is unique, depending on the bereaved person’s beliefs, experiences, temperament, and other factors, while the emotions felt may overlap and contradict one another (Callen, 2022; Avis et al., 2021). Each person experiences grief individually, taking as much time as needed and seeking a meaningful way to come to terms with the loss.

Society tends to view perinatal loss as less significant than the birth of a stillborn baby or the death of a loved one who was already born. “Instead, there is a culture of active denial and intellectualization that discourages parents from grieving” (Markin & Zilcha-Mano, 2018, p. 21). Early pregnancy loss is often referred to as a “silent” or “invisible” loss, as miscarriage is perceived as a “non-event” and the fetus as “not a person” (p. 21). Thus, women who lose a child in the early months of pregnancy may feel that their “grief is not real, should not be expressed publicly, and must be experienced in solitude” (p. 21). In reviewing grief studies, Markin and Zilcha-Mano (2018) found that turning to loved ones during a loss is a universal human reaction (p. 20). Since, in many cases, very few people know that a miscarriage has occurred, it becomes a “secret” grief that lacks cultural and social forms of expression (Seftel, 2020). Furthermore, it is very difficult to express this verbally, so the art therapy process becomes a bridge between the conscious and the subconscious. Malchiodi (2020), in defining the essence of working with traumatic experiences, identifies three main stages of art therapy: externalization, symbolization, and integration. In the process of externalization, those experiences and feelings that typically lacked the words to describe and name them can be given visual form through the art therapy process. During the creative process, abstract, vague experiences become accessible to reflection, as it is much easier to analyze visual, tangible elements—colors, shapes, symbols, and so on. Finally, by transforming and reflecting on one’s creative product—which conveys difficult experiences—a new relationship with the experience is formed, creating an opportunity to view the difficulties from a different angle. In this way, art therapy can function as a corrective experience, helping to embody the experience and reconstruct the relationship with oneself, the body, the environment, and the experience itself (McKernan, 2020). This process allows one to make sense of the difficulties and integrate them into one’s life. In Lithuania, if a miscarriage occurs very early, the fetus’s body is usually not recovered, so it is not possible to bury it and thus say goodbye and give meaning to the pain. Thus, in many cases, there is no farewell ritual, which is very important for the integration of a painful experience. Therefore, art therapy provides an opportunity not only to process difficult emotions and grieve in a safe environment, but also to symbolically say goodbye to the deceased child through drawings, letters, and dedicating works of art to them (Seftel, 2020).

**The Rationale for Art Therapy.** Art therapy in Lithuania is regulated as a healthcare activity falling under the category of psychosocial intervention services. The profession of art therapist and the requirements for practice are established by the Ministry of Health of the Republic of Lithuania, which defines the qualification standards and competencies for specialists working in this field (Ministry of Health of the Republic of Lithuania, 2018). According to current legislation, art therapy encompasses the fields of visual arts, music, drama, and dance-movement therapy (Ministry of Health of the Republic of Lithuania, 2018). To obtain the qualification of an art therapist, a master’s degree in the field of health sciences is required. In Lithuania, Vilnius University or the Lithuanian University of Health Sciences are authorized to offer such programs. One of the authors of this article completed these studies at the Lithuanian University of Health Sciences.

## 3. Justification for the Choice of Research Instruments

The aim of this study is to explore the experiences of women who have experienced a spontaneous miscarriage through the process of art therapy. Therefore, qualitative research methods were chosen, which are used to delve into phenomena and gain a deeper understanding of them. On the other hand, qualitative phenomenological research is particularly well-suited to revealing the experiences of this specific sample. Thematic analysis was used to answer the first research question. The data were therefore analysed using thematic analysis (Braun & Clarke, 2006, 2013).

In general, research of this nature is characterised by an endeavour to delve into the participants’ experiences, setting aside prior knowledge and judgement. All of this is important so that it is possible to view the phenomenon under investigation in a new light. Thematic analysis is a method of qualitative data analysis characterised as systematic work with qualitative data involving coding, identifying typical patterns and formulating related themes, with the aim of revealing the research issues (Žydžiūnaitė & Sabaliauskas, 2017; Kvale, 1983). The methodology of thematic analysis can be based on two approaches—the realist or the constructivist. This study adopts the realist approach, which focuses on the current experiences of the research participants and the meanings attributed to them within the participants’ real-life environment (Chang & Wang, 2021; Žydžiūnaitė & Sabaliauskas, 2017). It is argued that this method is particularly useful for topics that are sensitive or under-researched (Sezer & Aki, 2024; Žydžiūnaitė & Sabaliauskas, 2017). Our chosen topic is precisely such a subject. In thematic analysis, clear structuring of data is particularly important, as this allows for an exceptionally in-depth examination of the data, highlighting its characteristics, subtleties or complexity. The analysis and interpretation of results depend on the research perspective. Researchers applying this method rely on either an inductive or deductive perspective. This study adopts an inductive approach, as the aim was to reveal the authentic themes emerging from the participants’ reflections. Induction was also evident in the fact that the research was conducted first, followed by a detailed review of the literature. Prior to the research, the analysis of the scientific literature was carried out solely to develop themes and activities for the art therapy sessions. It is also important to emphasise that the themes for the art therapy sessions were developed following four supervision sessions, in consultation with psychologists and art therapists from Lithuania and abroad.

### 3.1. Study Participants

Four women who had experienced a spontaneous miscarriage took part in the study. They learned about the study from information posted on social media, as well as from staff at the Crisis Pregnancy Centre. This sample size was chosen for two reasons. Firstly, the research methods. As a phenomenological approach was chosen, the sample size is irrelevant (Žydžiūnaitė & Sabaliauskas, 2017). Furthermore, the sample size in qualitative research is not defined by clear criteria, as the adequacy of the sample is determined by ‘information power’ (Malterud et al., 2016). A researcher who chooses the phenomenological method is not concerned with the number of participants in the sample—they are more interested in the participants’ experiences. The result obtained is evaluated based on whether the sample selection method and the criteria for selecting research participants correspond to the research questions posed (Žydžiūnaitė & Sabaliauskas, 2017). Therefore, the following criteria were used when selecting research participants: adult women aged 20 and over, reflectivity—the ability to analyse and convey one’s experiences, and participation in all art therapy sessions. Prior to the study, all participants were informed about the research, and consent was obtained from each. Each participant signed the consent to participate and the informed consent forms. During the first session, participants were introduced to the aims of the study and the structure of the sessions, with an emphasis on the fact that reflections on the drawings would be recorded and used for research purposes. Furthermore, each participant was informed of their right to withdraw from the study at any time. Following the cycle of art therapy sessions, three main participants were selected for data analysis. This was because these three participants attended all sessions. Another participant attended seven sessions, missing two sessions at the end and one at the beginning. Such fragmentation of the therapeutic process would potentially limit the qualitative analysis, prevent certain themes from emerging, and result in a lack of data saturation. The participants were aged 33–43; all were women living in a large city, had attained higher education, three of them were married and living in a marital relationship, and one was unmarried. Two participants had experienced a spontaneous pregnancy loss 10–14 years prior, whilst the other two had experienced it within three months of the study’s commencement. Three participants experienced the loss during the first trimester of pregnancy, and one at 19 weeks.

### 3.2. Research Ethics and Organisation

The study was conducted between March and May 2020. The first two art therapy sessions took place at the premises of the Crisis Pregnancy Centre in Vilnius, but due to the prolonged lockdown, they were subsequently continued remotely. Permission to conduct the study was obtained from the Centre for Bioethics at the Lithuanian University of Health Sciences (approval registration No. BEC–DT(M)–1949). Ten art therapy sessions were conducted, each lasting an average of approximately 2 h, once a week; each session was recorded on a voice recorder, and the subsequent transcribed reflections lasted approximately 15 min. The themes, methods, objectives and tools of the art therapy sessions are presented below (see Table 1). At the start of each session, the participants’ well-being was discussed, along with any current events from the week they wished to share. This was followed by the presentation of the art therapy theme, based on which the participants created their work, with 30–40 min allocated for this. The participants then reflected on their creations. The reflections were recorded on a voice recorder, having first obtained their consent. As only 3 out of 4 participants attended all 10 art therapy sessions, the study analysed only the reflections of those women. The study participants were informed about the study, and their written consent was obtained for the analysis of interview recordings and drawings for research purposes, on the condition that their identities would not be disclosed.

As can be seen from the data presented in Table 1, various art media are presented, which the study participants are free to choose. It is important to note that the art media were chosen to convey and express their difficult thoughts, feelings and experiences related to the loss of a child. Drawing is a means of self-expression that helps one to open up about a very complex and distressing topic. The art therapy cycle covers the content of different days (Tekutienė, 2022). The study was conducted in Lithuanian, and all responses in the article have been translated into English.

The drawings created by each study participant during art therapy sessions are included in the appendices: Elzė’s drawings are in Appendix A, Jolita’s drawings are in Appendix B, and Gertrūda’s drawings are in Appendix C.

## 4. Research Results and Their Discussion

### 4.1. Thematic Analysis of the Experience of Elzė, a Participant in the Study

Elzė is 42 years old; her spontaneous miscarriage occurred three months before the start of the art therapy sessions. After starting to bleed, she was told at the hospital that due to a serious infection, labour would have to be induced; she was 19 weeks pregnant at the time. She has two children and is married. Elze attended all art therapy sessions. A thematic analysis of the data revealed the experiences she went through during the art therapy process. Analysis of the data revealed four themes that shed light on the experiences of the study participant, Elzė, during the art therapy process:

1. A difficult experience of hospitalisation.
2. ‘It knocked me for six’.
3. Acceptance of loss.
4. ‘I want to start living my life’.

The results of the thematic analysis of Elzė’s data (main themes and sub-themes) are presented in Table 2.

**Theme 1. The difficult experience of hospitalisation.**

**Sub-theme 1. Helplessness, fear and guilt.** Finding herself in hospital and realising the diagnosis, the woman experienced fear: “I think the most terrible feeling for me was fear. I was very, very scared” [crying]. The hardest thing was accepting that I had to obey and do what was essentially against my nature: “The worst thing is that I can’t change anything. That I can’t do anything myself. And what’s being done to me is something I don’t want, but I have to do it. For example, giving birth and thus being forced to let that child die. So, that helplessness is the strongest—they’re doing this to me, and I can’t resist” [sobs, sniffs], “there really was fear and pain, and this feeling that I can’t do anything, I can’t tell those doctors to get out, no, I have to endure it all and that’s it” [cries]. Another difficult experience—guilt, manifesting in thoughts about what else could have been done: “and then, after about a month— —I started thinking, why didn’t I do anything. Why didn’t I speak to the doctor? Why didn’t I ask for her to be put on a ventilator? Why didn’t I insist? Why didn’t I take that step? I could have changed something, but I did nothing. At first, I didn’t feel guilty. I thought, well, that’s just how it happened, it was God’s will. They carried out an examination of the foetus and, well, it wasn’t my fault. But after a while, that feeling of guilt set in, although I’m at peace with it now.”

**2. The doctors’ indifference.** Elzė encountered indifferent behaviour from the medical staff and received no offer of psychological support: “A nurse came and gave me medication to induce labour—that was it, she didn’t say a word.” The woman feels resentment that the doctors did not suggest trying to save her daughter’s life: “I feel anger towards the doctors. I started thinking, if others survive 21 weeks, why couldn’t mine at 19? Why didn’t they even suggest I try? And then, after we’d buried her, there was a news report on TV about a baby in Lithuania who’d survived to 22 weeks. It was the first case in Lithuania where a baby had survived to 22 weeks. And she weighed 400 g. And I was so happy then, thinking how wonderful it was that that mum hadn’t gone through what I’d gone through. But the doctors really could have tried, and maybe it would have…’ [she doesn’t finish her thought, she’s crying]. The woman was also surprised by the staff’s lack of empathy: “They showed no sympathy at all, they just went about their work. After the operation, I sat alone in the ward for about three hours. It was strange that no one came to see me… Well, somehow… Not even a psychologist.”

**3. Physical and emotional pain: “My body is about to burst.”** Elzė had to decide for herself whether to induce labour immediately upon arrival at the hospital or the following day. She decided to wait through the night, as she wanted to say goodbye and spend the remaining time with her daughter: “After the doctor examined me, she asked if I’d prefer to go through with it all today or tomorrow. Because it was clear that there was no way to save the pregnancy and I would have to give birth to that child. I’d need induction drugs. And somehow I still wanted to spend some time with her. Well, somehow. And that night… They brought me some sedatives, but I said I didn’t need anything. I wanted to bond with her, because I used to sing songs to her. Well, until then. I used to tell Jonas [my son] about Onutė [my late daughter]. And somehow I still wanted to be with her, I wanted to calm her down too.” The woman felt it was incredibly difficult to swallow the labour-inducing drugs; even her body resisted them: “And I can’t bring myself to take them, I’m scared, because I have to start that procedure myself. That was the scariest thing for me. Then I thought, maybe I just needed to wait for the birth to happen and that’s it. [crying] Besides, I really, really felt that my body was resisting. I started having these convulsions. I’ve never felt anything like that in my life. My whole body was shaking.” Comparing her previous births to this one, Elzė notes that the latter was significantly more complicated, even though, in theory, it should have been easier to give birth to a baby who weighed considerably less: “Then that birth was very difficult. I gave birth to my son Jonas more easily than to Ona. It felt as though my body was about to tear apart; the physical pain was excruciating, it’s hard to explain [sobs, cries]. And then the placenta didn’t come away; they had to pull it out of me, it hurt unbearably. Then, finally, the bleeding started, and they performed an operation. It was all so frightening.”

**4. Loneliness and efforts to protect her husband.** Although the woman consciously chose to give birth alone, refusing to have her husband present, she experienced pain, fear and loneliness: “And I was alone, but that was my choice. I thought I’d get through it all on my own, I thought my husband would react very sensitively, and I thought I’d manage on my own, but it was actually terribly hard for me. I don’t know [pause] There really was fear, pain.” The woman only told her husband about the complications of the birth a few days later; it was important to her to spare him from the emotional strain: “I couldn’t tell my husband because he gets very stressed when he sees me suffering or feeling sad. I thought, I won’t tell him everything that happened; I’ll just say they took it out. And he came just to pick me up; after everything, after the operation, he came to pick me up. And for two days I didn’t tell him anything, I didn’t say a word. But then somehow I couldn’t hold back any longer, I…’ [cries, unable to finish her thought].

**5. The hurtful attitude of other people**. While in hospital, the woman encountered a different attitude from other women in a similar situation. Her wardmate’s story caused Elze pain: “I’m sitting there and crying all the time. And she asks me, ‘Is it the same for you as it is for me? I say, I don’t know what’s wrong with you… and she tells me—they found a deformed baby inside her… can you imagine, a deformed baby has grown in my womb. ‘So what, you’ll give birth to another one—a healthy one.’ I tell her, ‘Here’s your child. “What kind of freak?” I tell her, “No, it’s definitely not the same for me. For her, it’s more like ‘hurry up, hurry up ’ to get that freak out, but for me it’s completely different…” On the other hand, having already buried her daughter, the woman faced pressure from those around her to pull herself together and carry on living as if nothing had happened. The woman sensed this from her husband, but tries to excuse him: “Well, let’s say, my husband, he really went through it all very deeply, and somehow time passed and he stopped talking about it. He stopped. He says, ‘Come on, pull yourself together, enough is enough. And that sort of annoys me, but I understand that he can’t bear it either. He didn’t give birth, he wasn’t pregnant, you have to understand him too, don’t you?” However, the woman took a clear stance on talking about the loss: “I actually wanted to talk about it. It was important to me.”

**Theme 2. That ‘knocked me for six’.**

**Sub-theme 1: ‘The feeling that my life had fallen apart’.** After the induced labour, the woman felt that her life had fallen apart: ‘I lost her… [crying]. Because it feels like my whole life fell apart then’ [pause, crying]. The woman describes herself as positive, having been through a lot, but this event broke her: “And all my life I’ve been a strong person, and this thing just knocked me for six [sobs, cries]. <…> I always smiled a lot, laughed, was so happy. And everyone really did describe me that way. But now, completely…” [crying].

**2nd theme. The collapse of her greatest dream in life—to have a daughter.** Such intense emotions are also driven by the fact that Elzė’s lifelong dream—to have a daughter—has been shattered: “I’m 42. I’ve dreamed of having a daughter my whole life. My whole life. And when I got pregnant, when I found out it was going to be a girl, everyone was so excited for her, everyone knew how much I wanted it.” The woman experienced a startling, unexpected realisation that she was not all-powerful: “Well, I just didn’t expect that this could happen. Well, actually, the whole time, my pregnancy was incredibly pleasant. The feelings were very pleasant; I was really looking forward to it, I really wanted it. It’s been like that since childhood. I’ve always wanted a girl. But I have two boys. It was all incredibly unexpected. I didn’t think it would be like this. My dad used to tell me, ‘Just look after her, you know.’ I’d say, ‘Dad, trust me, now that I’ve got her [strokes her bump], I’m all set, I’m invincible,’ and it turns out I’m not… [cries].”

**3. Hiding her pain: “My smile is fake, my cheeks are sunken.”** Elzė feels an inner pressure to ‘pull herself together’, not to show her true emotions, because she is raising a pre-school-aged son at home, whose behaviour has begun to regress following the loss: ‘He’s acting strangely, I don’t know, he’s just… [sniffs]. Still, my little one is at home. He feels it deeply. He stopped talking. He stopped talking and then he started wetting his trousers again. He’s taking all that grief from me and absorbing it. Seeing what’s happened, seeing how he feels, I stopped crying in front of him. Well, he’s having his afternoon nap, so I can hug a pillow and have a good cry [and she laughs and cries]. And just then he walks into the room and I immediately go, ‘Oh, oh, everything’s fine, nothing’s happened here.’ The woman experiences mood swings: “Mood swings are also when the sun is shining, and I feel like turning up the music on the stereo. Just like that! [shouts, speaks with passion, enthusiasm]. Then suddenly it’s back again. It’s like waves. It’s very hard. I want to scream, to cry.” Elzė, watching herself in the mirror, notices changes in her face: “I recently came up with this idea of going to the mirror and smiling at myself. And I stand in front of the mirror and it seems to me that my smile is artificial. And my cheeks, which have to hold that smile, they’ve atrophied. It’s hard for them to hold the smile because they’ve atrophied [she pinches, squeezes her cheeks].’

**4. Psychological trauma symptoms.** The woman suffers from sleep disturbances and recurring visions of childbirth: “I can’t sleep at night. I wake up a hundred times.” Elzė sees her daughter in her dreams, recognises her features in other children: “I dream, I see her little face. Well, just like that. And…” [cries, does not finish the sentence]. <…> You get drunk and then, well, it’s easier not to think about it, but one night I just dreamt of her and woke up feeling all… well, all like that [speaks slowly, disjointedly] and it started all over again. <…> And another strange thing is that, when she died, I dreamt of her very clearly, very clearly as a 3- or 4-year-old girl, just as I’d seen a very similar child online, well, incredibly similar. And I was so confused.”

**5. Tension in the body and trying to help herself.** Elzė feels certain symptoms in her body, which led her to see doctors: “I went to the doctor and they said everything was fine, but I still feel it.” She feels discomfort in her throat, chest and lower back: “I don’t know, there’s this lump in my throat all the time, and in my chest there’s this slimy slug living there, and in my stomach and on the lower back side, it’s like my skin is being torn.” She also describes sensations in her stomach: “There’s a bit of a rumbling there.” And in her head: “There’s a kind of throbbing in my head. Like a void. That’s what I feel when I listen to my body. And that throbbing—it’s not pain; it’s constant, and I wouldn’t even call it pain. It’s like some muscles are tensing up.” Elzė listens to her body, doing so in an attempt to understand what might help her overcome these sensations: “And that tightness, it stops me from breathing, and then there’s my throat. It feels like I’m constantly short of breath. I lose it when I go for a run—it’s been a couple of weeks now—and when I come back from running, neither the lump in my throat nor that phlegm is there.” Still, the unpleasant sensations return: “I wake up the next morning and there it is [the lump in my throat] again. For me, it’s linked to breathing; when I run, it clears up, but that nasty thing comes back. Sport helps me somehow, but…” The woman notes that the opportunity to talk things through also helps her: “Again, perhaps it’s that need to talk things through; I feel that it’s still there. I’ve spoken to my dad, I don’t want to talk to anyone else, but I can only talk about it in therapy. <…> And I feel that when I talk about it, I feel better. When I’ve spoken to you, I really do feel better that very evening. And running is like a state of meditation for me. My thoughts become clearer when I’m running.”

**6. Reduced need for social interaction, rejection by loved ones.** The woman speaks of a changed relationship with her friends: “And over the last few years, a lot of friends have drifted away, with whom I had an unclear relationship.” She is experiencing rejection from those close to her: “My friends start avoiding me when I talk to them about it.” On the other hand, the woman herself feels a reduced need for social interaction: “Lately, I’ve been socialising very little with people.” She doubts whether her experiences matter to others: “Who’s there to tell? Who’s there to talk to? Your problems might seem unimportant to them.” Elzė emphasises that in the therapy group she can feel accepted and important; she also singles out her father—she opens up to these people: “I drew you closest because I can be open here”, “I can only tell everything and talk to my father and to you.”

**Theme 3. Acceptance of loss.**

**Sub-theme 1. Expressing grief through the need to talk about the loss.** The woman allowed herself to grieve. She cried as much as she wanted: “I didn’t care, I cried as much as I needed to, and I still do that sometimes.” She listened to her own needs: “I try to do what I really want, so that there is no coercion.” Elzė spoke about her pain; it was important to her that people also heard and acknowledged what had happened: “Some of you said you didn’t want to talk about it, but I actually wanted to talk. It was important to me that people didn’t act as if nothing had happened.” She allowed herself to accept the loss, to come to terms with it: “And so I’m free to say that I’m depressed, that I’ve lost a child and that I’m not well, that I’m coming here and seeing a psychologist. And I’m fine with that, because I’m helping myself.” Despite the fact that she could not share her experience with everyone, the opportunity to share her feelings was very important to her, easing her suffering: “I’ve definitely calmed down; at least I’ve found a space where I can talk about it, and that’s very important to me.”

**2. The peace she found visiting her daughter’s grave.** After the funeral, the woman often went to the cemetery. This evoked difficult feelings in her: “I’d go there and feel as though I were falling apart. I’d cry buckets.” However, after four therapy sessions, she felt differently: “And I was at the cemetery just now, and I felt so calm. It was so beautiful… So many blue flowers. And today we went there with the child. We just sat there in the cemetery, just sat there, and it was just absolutely lovely,” “Now, when I went there, there was a bit of sadness, but not overwhelming; really, I felt calm, I no longer associate myself with all that pain; such peace has come, even though there is still some sadness.”

**3. A turning point through the interpretation of significant symbols.** The woman links the improvement in her state to an event where, during one of the therapy sessions, a seed planted in the ground bloomed on the very day her daughter was due to be born: “The turning point came when we planted the flower and it bloomed just as Onutė was due to be born. It was almost mystical. Some kind of cosmic thing. So powerful. I felt such a sense of relief then.” This physical act helped to give meaning to the loss: “For me, planting that flower and watching it grow was so special, as if that was the meaning.”

**4. Letting go and accepting one’s situation**. During the art therapy process, the woman realised that the time had come to let go of the pain: “I feel that it’s time to say goodbye, well, not goodbye, but finally let her go. [Pause, ponders] Peace is what I feel now <…> Well, of course, that letting go, you know. She will always remain in my heart, but now I am at peace, even though I am still sad.” This brings Elze relief: “Well, I’m letting go of that pain, well, sort of, I suppose, and I feel that life is definitely easier for me now.” The woman finds meaning in the pain: “I believe that something should grow out of that little bit of pain, don’t you think? <…> I believe that pain changes people, and it always changes them for the better. That’s why you have to accept it, no matter how painful it is; in short, thank you all so much, and nothing happens for no reason.” Making sense of her loss is important to the participant, so she tries to discover how to do that, and her faith becomes her pillar: “And I keep wondering what I’m meant to understand from this, what will come of the fact that I’ve lost Onutė. I keep waiting and wondering what’s going on here, why this is happening. I believe that nothing in life happens by chance.”

**5. The importance and influence of the art therapy group.** The art therapy group was the place where the participant found help, acceptance and a listening ear: “I’m glad I received this help. Here I found understanding and a listening ear.” Getting involved in the art therapy process helped the woman bring clarity to her life and find peace: “Because before you came along, it felt to me as though the end of the world was coming. But when you arrived, everything started to fall into place.” For this reason, Elzė feels grateful for her restored resources and her will to live: “And I am truly very grateful. I feel truly immense gratitude towards everyone, because I found out about this therapy at the very hardest moment, and now I’m crying because I feel such deep gratitude, as it really, really helped me get through everything and start living again [crying, smiling].” The art therapy process helped Elze get to know herself better and discover new things: “Through this therapy, I’ve identified more than one thing that’s very important to me and, in general, gained a deeper understanding of myself.”

**Topic 4. ‘I want to start a new life’.**

**Sub-theme 1. Efforts to create her dream job.** In subsequent therapy sessions, the woman begins to talk about her changed attitude towards work. She feels ready to start working, and not just any job, but the one she has been dreaming of: “I used to say that I couldn’t change anything. But now it seems to me that work might help. And the day after tomorrow I’m going to that thing of ours. Well, that’s how it’ll be, that’s the selection process. I don’t want to talk about it because I’m scared it won’t work out and I don’t know if it’ll happen. I thought the time had come. I’d been dreaming, and then someone rang me and offered me the chance to do everything I’d planned out in my head. Honestly, it’s like a dream come true.” The woman explains that raising her son, then expecting her daughter, and going through a loss took a lot of energy, so she wants to make up for it: “I devoted all this time to my son and read books on parenting, then this incident with Onutė—that’s how I lost a part of myself.” The woman believes that this offer she has just received is a sign that it is time to gradually move on from the grieving process: “And so I’m feeling anxious and really looking forward to it and getting ready, and thinking about it a lot. This is the phase I’m in right now. And everything just fell into my lap. And maybe this is a sign that I need to move on, to do something to move forward. I feel like this is my chance. <…> And everything’s churning inside me, well, like this” [she makes a twisting motion with her hands]. These new thoughts about her dream help divert her attention from the pain; she feels ready to embrace change and new beginnings: “And somehow I feel that this work takes my mind off the pain a bit. I think more about that than what happened to me. If that offer had come a month or two ago, I would have cancelled everything, but now I’m really looking forward to it, I want it and I’m ready.”

**2. Practising new activities.** During the therapy process, the woman sensed and identified what might help her get through the grieving process. So she started going to the swimming pool: “I’m just so glad I’ve started swimming.” This activity helps Elze relax and calms her: “I’ve already told you that when I go to the pool, get into the water, I realise that this is what will help me get through it. I relax completely, and I’m a different person.” She also started running: “It’s been a few weeks since I started running again. Running is like a state of meditation for me. My thoughts become clearer as I run. I feel so clear-headed, so strengthened.”

**3. The fear of having another baby has gone**. In later sessions, Elzė noticed a change in her feelings and attitude towards the possibility of trying to have a baby again: “I realised that quite recently I stopped being afraid. Afraid of having a baby.” The woman realises that the outcome of every pregnancy is different; she no longer dwells on the idea that if it happened once, it will happen again: “And if it does happen, well, that would be a completely different story. And a different outcome, most likely—I also see that as a sign of healing. Because until then I thought it definitely wouldn’t. And I used to say for years and so on, but somehow, around those years, I think that if God grants that child, then he will be born. My mum gave birth later in life. Everyone has their own time.”

Summarising the data from the thematic analysis, several of the most striking experiences of the study participant Elzė during the art therapy process can be identified, which are presented below (see Table 3).

In summary, it can be said that, first and foremost, the woman faced a profound sense of helplessness, which she felt whilst in hospital, doing as the doctors instructed. They showed no empathy; to Elze, they seemed indifferent, showing no compassion whatsoever. The woman recalled the terrible physical and emotional experiences associated with being forced to take medication to induce labour and the birth of a stillborn baby. This experience caused a sense that her whole life had collapsed. This led to insomnia and other symptoms of psychological trauma. Another important aspect was the feeling of loneliness, incomprehension and rejection, and the confrontation with the hurtful attitudes of others. Nevertheless, during the course of the therapeutic process, the woman discovered the resources to accept the loss, make sense of it, and allow herself to build the life she had dreamed of. The woman’s sincerity, her strong emotional engagement in the art therapy process, allowing herself to grieve, processing her emotions, and talking about her pain helped her find peace even when visiting her daughter’s grave, to start pursuing her dream job, to take up new activities, and to entertain the thought of trying for another child.

### 4.2. Thematic Analysis of the Experience of Study Participant Jolita

Jolita is 38 years old; she suffered a spontaneous miscarriage 16 years ago, which she discovered whilst at the doctor’s, and a curettage was performed. She lives alone. Jolita attended all art therapy sessions. The participant’s statements were recorded and later transcribed. The thematic analysis of the data revealed the experiences the woman went through during the art therapy process. Four main themes were identified.

Analysis of the data revealed four themes that shed light on the experiences of the study participant, Jolita, during the art therapy process:

1. Allowing herself to grieve.
2. ‘Life has simply disappeared’.
3. ‘Well, what? There’s no self-worth.’
4. The process of coming to terms with loss.

The results of the thematic analysis of Jolita’s data (main themes and sub-themes) are presented in the table (see Table 4).

**Topic 1. The uncontrollable grieving process.**

**Sub-theme 1. Self-blame**. The woman feels a great deal of guilt over the miscarriage; she describes it as follows: “I wasn’t able to carry it to term”, “I blamed myself for a long time, thinking I could have done something, worked less, taken more care of myself; why didn’t I rest, why did I rush about everywhere?” She wonders why she did not consult several doctors, why she trusted one specialist’s diagnosis and immediately agreed to an abortion: “And why didn’t I go to fifteen doctors, why didn’t I wait until my body rejected it itself, because if it’s a mistake, then it’ll reject it itself” [crying, shouting]. Jolita compares herself to a woman she read about in the press: “And, f***, that woman they told, like, it wasn’t developing, is now raising a child of her own.” The woman finds it hard to understand why the pregnancy ended: “If the tests didn’t show anything wrong. No inflammation, nothing wrong, [crying] so why was it wrong then?” Jolita also feels guilty because she never spoke to anyone about it and did not allow herself to let it out: “That was my mistake. I admit it. [sobs] You always need to cry and talk for as long as you want… [sniffs], because, as experience shows—14 years have passed, and I’m still crying, and I haven’t spoken to anyone.”

**2. Withdrawal and isolation.** Jolita decided not to share her experiences with anyone because she felt misunderstood; since those around her, having neither experienced nor gone through similar hardships, simply cannot understand what it means to lose a child: “I was very, very cold, closed off, and couldn’t talk to anyone.” Feeling that others might not understand her, she wanted to shut herself off from the outside world: “I thought, why tell anyone? They wouldn’t understand anyway. That’s why I didn’t say anything. It was easier to be with myself than to explain things to someone else.” After finding out that the pregnancy was not progressing and following the D&C, Jolita split up with her boyfriend at the time. The reason was his lack of understanding: “The conversation went like this: I’d had a D&C, he knew, but he didn’t even pick me up from the hospital because he was supposedly working so hard. He even asked why I was so quiet. [laughs sarcastically] That’s when I realised he was absolutely thick. After that day, I realised I couldn’t see a future with him” [cries].

**3. Denial of the fact of loss**. The woman no longer wanted to remember or try to come to terms with what had happened; she decided to distance herself from the traumatic experience. As she was working and studying at the time, she threw herself into her activities and told herself: “It happened to me. Fact. That’s it. Full stop.” For a while, the woman still tried to push the bad thoughts away, but from that point on she stopped crying: “I used to tell myself, ‘Go on, have a good cry,’ but no… Nothing happened,” “I should have cried, but at the time I felt nothing. Nothing.” She compares this detachment from her own feelings to a closed door: “I drew a closed door here; now, through therapy, I’m opening it. Everything is pouring out, overflowing. I’m afraid to open it all the way, and I wonder if I’ll manage. But I’m definitely opening it right here, behind that handle.” Despite the fact that she did not consciously let it all out, she sometimes felt bad for no apparent reason: “But that pain… But everything always comes out later. You just don’t know why you’re constantly crying. But that’s how it was, because I didn’t cry it out, I didn’t let it all out.”

**4. “Bubbling” feelings.** The woman recognises a multitude of different emotions associated with the loss and the state following it. She states that it is mostly anger: “I was terrified that there was so much of it.” The research participant uses various descriptions to describe her multifaceted state: “To put it simply, saying I’m just angry isn’t enough, because I’m disgusted by what happened. It’s rage, and betrayal, and jealousy, and rejection, and bitterness—I don’t even know if I can list them all; it’s hard to put into words. And it turns out there was a lot of sadness too. And furious, abandoned, and helpless, grieving, and empty, and disappointed.” The woman says there are so many feelings that she finds it hard to process them: “It’s unreal how much I’ve felt and haven’t felt; only now am I realising that it’s all built up.”

**5. Tension in the body**. The woman identifies three places in her body where she feels tension: “There’s the stomach and part of the back—the lower back and the head.” The sensations there are not constant: “Sometimes I feel something loosening up there, and then a second later it’s back again. There’s also tension in my head.” The participant states that the cause of this tension lies in her thoughts: “If I thought less, it wouldn’t be there—my brain is constantly tense and I’d so much like not to think about anything, so that it wouldn’t be like that anymore.” Jolita also mentions “strange” sensations in her heart, which are linked to the birth of children in her immediate circle: “The tension is there when someone gives birth, whether in my immediate circle or elsewhere, and it always passes through this part [she gestures with her hand around her heart area].” She emphasises that she cannot cope with these tensions: “I can’t do anything about it. I don’t even know if it will ever go away on its own, or if it will ever go away.”

**Topic 2. “Life has simply disappeared.”**

**1. Failed expectations.** Jolita dreamed of her future baby: “The baby was very much wanted and I was so happy.” She often thought about what it would be like when the baby grew up: “Every now and then I’d think about how I’d take the child to the nursery near our home. I had really projected myself into that and imagined that this would be my life.” Because of her shattered dreams and future plans, the woman felt that: “life had simply ceased to exist.” The experience of loss was so intense that she could no longer appreciate what she had: “It feels as though everything fell apart that day, even though perhaps it shouldn’t have.”

**2. The mismatch between her desires and how she lives.** It was hard for Jolita to accept that life was not at all what she had wanted: “I don’t know what I’m actually doing. I’m working here, building a career, but I don’t really have what I wanted most [sobs]. Why am I here, all tough and tough-talking, driving around in the best cars, able to have whatever I want, but those aren’t the things I want [cries].” For this reason, the woman experienced a sense of meaninglessness: “And then it’s like, what’s the point?” Comparing herself to other women only intensified this feeling: “Well, for example, a colleague goes off to give birth, and then the work feels so pointless, what’s the point…”

**3. The indifference of those around her: “Maybe I’m adopted after all?”** After the D&C, the woman returned from hospital on her own, as none of her close relatives could drive her home. This caused a feeling of loneliness, abandonment, and anger: “I felt so alone”, “bloody hell, I was completely on my own” [crying] <…> and no one, absolutely no one, could come. My parents, well, they lived far away, but still, this isn’t just any old event. I felt like an orphan.” Because of this indifference from her loved ones, the woman began to wonder if she was adopted: “Actually, I even started thinking, maybe I’m adopted after all.” Because of these experiences, the woman belittles herself: “I felt like such a fool, a failure.”

**4. Unresolved feelings complicating relationships.** For many years, these unresolved feelings would erupt in inexplicable outbursts of anger directed at the people around Jolita: “Now I understand why I’d suddenly fly off the handle for no reason at all, just because someone asked me something.” The woman often felt very sensitive and vulnerable: “Someone would say something, and for me everything would just triple or double; I’d react so exaggeratedly.” Unresolved feelings prompted the participant to put on “armour” and be “cold, unapproachable”: “Well, there I go, just leave me alone, don’t talk to me.” The woman longs for peace: “And then, after painting so many colours with those feelings, I realised that this is truly the cosmos. There’s just so much of everything. It’s unreal. I started to realise that I want peace inside, then I won’t snap, I won’t be critical; what difference does it make how that person looks today, I accept that, I see it, well, the thing is, there’s so much anger that if someone just says something, I’m like, ‘waaaaah.’”

**5. A frightening emptiness.** After losing her baby, the woman began to feel a recurring state—a sense of numbness: “Well, just this empty feeling. Maybe indifference… [pauses, falls silent] <…> I just go on and that’s it, so numb.” This causes Jolita fear: “and that path is my fear of walking into the void”, “I’m afraid of feeling nothing”, “like a corpse, lifeless.” The woman describes this experience as the hardest to bear: “It was more terrifying than anything to feel nothing.” When experiencing this sensation, the woman would try to help herself cope: “And anyway, I look for something to do so I don’t feel it.” Nevertheless, the woman fears that this feeling might return; that fear triggers a sense of being haunted: “I’d describe it as a kind of darkness following me. That darkness lingers.”

**Theme 3. “Well, what? I have no self-worth.”**

**Sub-theme 1. “Why don’t they love me—‘I’m as useless as a whistle on a windy day’.”** The woman often experiences a feeling of being unloved. She feels the reason lies in her relationship with her mother: “But it goes back to my childhood, because I really did feel unloved.” Jolita is distressed by her constantly breaking-up relationships with partners: “… but he loves me, he doesn’t love me. Does he not love me? Why, why, why doesn’t he love me? [speaks emotionally, gesticulating with her hands].” The woman realises that she has a subconscious fear of letting go and allowing herself to be who she truly is at that moment, of showing her vulnerability: “And I think that if I don’t do something, he won’t love me. When I run out of energy, and I’m lying in bed, tormenting myself, feeling completely worthless, and thinking that I’m so uncool, so useless. <…> And really, I mean, I think that’s why he doesn’t love me. And I do everything, and then I run out of strength or some cycle kicks in and when I really…”

**2. Theme. Unstable self-esteem.** Jolita realises that the concepts of ‘self-confidence’ and ‘self-esteem’ are different. She describes the difference as follows: “I have self-confidence; I’m fine at work, I feel confident there, but in relationships—no.” The woman would like to be herself, not afraid to show her different sides, when interacting with everyone: “That’s my goal—to have the same self-esteem with everyone, because, for example, my friends accept me just as I am; I can get angry, but with my partner I have the lowest self-esteem. My awareness and common sense just switch off then.” The reason for such fluctuations in self-esteem may be a failure to forgive oneself: “But really, I’ve realised that I have to forgive myself first, and then everyone else, because I’ve certainly messed up plenty of things.”

**3. The desire to distance oneself from women who have children.** The fact that a woman does not have children makes her feel incomplete: “Sometimes I think I’m so incomplete.” This feeling prompts her to compare herself with other women who have children, to look for reasons why she cannot conceive: “Then you look for an explanation as to why there aren’t more children, why I haven’t been given the chance, whilst others have. The doctor explains that I’m healthy, I could get pregnant today, but if it’s not meant to be, it’s not.” For this reason, she feels the urge to distance herself from people who have children: “Then you become cold, distant, and those children, as if on purpose, stick to you like a magnet” [she cries].

**Theme 4. The process of coming to terms with loss.**

**Sub-theme 1. Realising and making sense of the loss by giving a name.** The woman’s state of mind began to change after she gave her unborn child a name: “Monika had suggested a name. And I just went along with it. The name came to me. And somehow everything changed for me. That’s it.” This provided an opportunity to make sense of and come to terms with the loss: “This thing gave it great meaning. There was no longer any denial. It’s sort of like, he’s certain that he was that son. [smiles] Because before that, it was so unclear, almost like a figment of the imagination or something.” Giving the name helped Jolita to internalise and acknowledge the fact of the baby’s existence: “I can tell someone else about it as a fact. Because before that it was: What? What happened? What do you mean? No, no, I really didn’t have anything.” This experience prompted the woman to write her son a story—a letter in which she told him about her current life, writing down what she wanted to say: “The most important thing is that you’re happy and that you have good parents up there in heaven, and anyway, I believe you meet your deceased relatives there; it’s good for you there. And I’m happy too. Really” [smiles].

**2. Discovering the positive aspects: “And I realise that I can’t change this situation, and through it I’m becoming a stronger person.”** The woman’s attitude towards her loss has changed: “I think you shouldn’t ignore it, but let this fact into your life”, “misfortunes are sometimes necessary too; for some reason, they have to happen”. Having worked through her difficult emotions, she began to discover the positive things in her life: “In a way, you know, I’m glad it happened, because I’d definitely be a single mum now. Because I really wouldn’t be able to cope. Now I’d be going to some other kind of therapy—on how to raise a child—because I’d definitely just be screaming, breaking down, and it would be much worse” [laughs]. In her loss, the woman finds strength: “Perhaps a bit selfish, but I thought it makes me strong.” Reflecting on how her life might have turned out if the baby had been born, Jolita realises she might well have been unhappy: “After that incident, I split up with my long-term partner, went on to study further, and a few years later became a headteacher. But if it hadn’t been like that, I wonder if I’d have ended up completely downtrodden, stuck with such a wretched, drinking husband.” Recalling a conversation with her aunt also prompted the woman to reflect on a possible scenario for the continuation of her relationship with the father of her unborn son: “I remembered that everyone was really against me seeing that man. Now I realise that my aunt said everything was fine here. God didn’t know how to separate you from him, so at least this is how you parted ways.” Such realisations help Jolita come to terms with things: “And I understand that I can’t change this situation, and through this I’m becoming a stronger version of myself.”

**3. The pain leaves me, stays on the pages.** Jolita finds that drawing calms her down; she feels the pain lessen: “I’ve been painting at home all week. It’s such a buzz, I feel really, really good,” “On Thursday, instead of attending classes, I painted again and posted it on Facebook; I feel as though the pain is leaving me, staying on the pages, and I’m tearing them up.” The woman discovers that by transforming her creations through the art therapy process, she also changes how she feels: “I felt that mmm [pause]… every pain or person, or grudge… I place it on a cloud and let it go. And after painting that, I really did feel that way.” This gave her self-confidence: “It’s cool, I felt kind of powerful.” Discovering art materials she liked brought her joy: “I started to really like watercolours. I bought some good ones, and that makes me happy.” The art therapy process inspired the woman to paint a picture depicting a woman with a baby, as a way of making sense of and coming to terms with her loss: “I want to create a painting, but not right now, because it takes time, but I know it’s definitely time now; I’ll share it on Facebook, so you can see it.”

**4. The importance of the therapy group.** Upon learning that a lockdown had been announced, the woman was disheartened that the therapeutic process would have to be put on hold: “It really was sad; I’m glad Monika asked us if we wanted to continue our group. I also want to tell you about a dream. Well, it was a really cool dream [she smiles, recounting it enthusiastically]. We’re all there. It’s light outside. Most importantly, we’re all together, and the sun was shining; I don’t know, I can’t make out where we are. And we’re all sitting in a circle, leaning in, telling stories, telling stories. And then that day, you, Monika, wrote that we’d continue with online therapy. I was so happy.” The woman says that during this period, the women in the therapy group have become very close to her: “You’ve become so important to me.” She feels grateful that she was able to open up, work through difficult emotions, and accept her situation: “Here are those doors; I open them and all the pain pours out through them. Just like now,” “I’ve opened up to you so much; it feels like I’ve let it go. The pain is gone now. It’s thanks to you that this is the case.”

Thus, the data from the thematic analysis revealed Jolita’s multifaceted experiences during the art therapy process (see Table 5).

First and foremost, the woman realised that she had not acknowledged the painfulness of the loss, had not allowed herself to grieve, and had therefore suppressed all her difficult emotions. Through therapeutic work, she recognised and processed feelings of guilt, anger, disgust, emptiness, loneliness, betrayal and numbness. Jolita recognised that her failure to grieve and the suppression of her emotions had affected her relationships with those around her, as it had caused her to want to distance herself from people, especially those with children. Furthermore, the woman realised that, despite the pregnancy being short-lived, she had dreamed of and planned a future life together with the child; consequently, the loss caused a sense of life’s collapse, a feeling of meaninglessness and inadequacy. Overall, the theme of self-worth frequently arose for Jolita during the art therapy process. The participant recognised that in life she often faces situations where she feels unloved and unaccepted; in some relationships, she cannot fully be herself and has to make a great deal of effort not to reveal her shortcomings. On the other hand, the name given to her unborn child, and the realisation that the loss had profoundly affected Jolita’s life, led to reconciliation and the discovery of positive aspects of her current life situation. Painting with watercolours helped her to overcome her difficult experiences. The support and sense of security found in the therapeutic group, along with the feeling of closeness experienced with the other participants, contributed to the process of transformation, the realisation of the loss, and the finding of meaning in it.

### 4.3. Thematic Analysis of Gertrūda’s Experience

Gertrūda is 33 years old; she suffered a spontaneous miscarriage 10 years ago, which she discovered during a doctor’s appointment, and underwent a D&C. She is married and raising two children. The woman participated in all art therapy sessions. The participant’s statements were recorded and later transcribed. The thematic analysis of the data revealed the experiences the woman went through during the art therapy process.

Analysis of the data highlighted two themes revealing the experiences of the study participant, Gertrūda, during the art therapy process (see Table 6):

1. The spectrum of difficult emotions in relationships.
2. Making sense of loss.

**Topic 1. The spectrum of difficult emotions in a relationship.**

**1 Sub-theme. The centrifuge**. Upon learning that she had suffered a spontaneous miscarriage, Gertrūda felt: “as if I were being tossed about in a centrifuge.” The image of the centrifuge aptly illustrates the woman’s psychological state and the difficult experiences she was going through. She felt powerless, unable to influence what was being done to her: “I felt helpless; the doctors explain everything, and I just do as I’m told,” “I felt as though everything was over for me,” “the feeling that I couldn’t take it any longer.” The experience of hospitalisation evoked a sense of objectification, insignificance and worthlessness: “I was just an object being tossed about and battered like in a centrifuge”, “that’s how I ended up as this cowering woman.” This was compounded by physical sensations: “I felt so sick, it was so awful.” The woman experienced intense feelings and emotions, such as: “Fear. The most terrifying moment was when they told me. I started crying right there,” surprise and “it was a shock to me; I didn’t expect it to be like that,” “I felt so vulnerable.” Furthermore, the woman experienced an overwhelming sense of emptiness: “Such emptiness, and it went on for so long.” The sudden and rapid sequence of events prevented the woman from realising or comprehending the loss; she felt as though she were “in a trance”.

**2. Not allowing oneself to grieve, the desire to be strong.** Upon returning home after the curettage, the participant suppressed any feelings related to the loss and decided not to talk about it with others: “I didn’t want to talk to anyone; I told my husband not to even speak to my mum about it”, “I just ran away from the subject”, “it feels like I haven’t really come to terms with it yet.” Due to her reluctance to communicate and share her experiences, the woman began to feel isolated: “it’s like shutting myself off”, “I feel as if I’ve shut myself off.” The woman tried not to think about the loss: “I didn’t feel that I was unwell, and if a thought did come to mind, I simply wouldn’t dwell on it any further and that was that.” Gertrūda did not let anything in; she describes her state of mind at the time as: “it was like being in armour all the time.” Nevertheless, having gone through the abortion, the woman did not conceive for four years, but she did not feel bad about it: “I didn’t cry, I was very positive; after all, I was building my career, I tried not to get stuck in a rut.” She only felt anger, pain and other difficult emotions once she began to look within herself and meditate: “I sit, I meditate, and the tears come; I realise I haven’t let go, that I’m carrying that little one, Viktorija (the name she gave her unborn child), the one I’d dreamed of—and then I realised there were issues, and then came this group.”

**3. A toxic relationship with herself, like a meat grinder: “you get stuck and spin round”.** The woman identifies several things that hinder her relationship with herself, which may be why she did not allow herself to grieve. Gertrūda realises that she usually does not feel satisfied with the current situation, so she constantly strives for perfection: “It’s a proper meat grinder, you get stuck and spin round, it’s not right, it’s not right, improve, improve.” To this day, the woman feels guilty about her reaction following a conflict with her husband, which occurred whilst she was still pregnant—excessive self-pity, which, in her view, may have contributed to the miscarriage: “Then I cried like that for a whole week—I felt so sorry for myself,” “I shouldn’t have fallen into a victim mentality, <…> but it was so sad.” She also recognised that the urge to blame herself is, in general, a common reaction of hers to various situations: “Well, I often blame myself for everything.” The study participant often berates herself over various things: “I’m always telling myself off—you shouted again, you did something wrong again [pause, ponders] I’m always thinking something bad about myself. That’s how you end up with such a toxic relationship with yourself.” Excessive expectations and demands on herself prevent her from feeling joy: “I always want to improve something, it’s always wrong and wrong,” “mindfulness, mindfulness, practice, I just can’t say ‘enough’.” In later art therapy sessions, the woman reflected that she tends to bottle things up and is reluctant to talk about them, but having recognised this, she is trying to change her behaviour: “When it all builds up, I keep it all inside, I don’t speak, I shut myself off, but I don’t want it to be like that anymore; I know I’ll let it out.”

**Theme 2. Making sense of loss.**

**Sub-theme 1. The importance of relationships and the meaning of life discovered.** Following the termination of her pregnancy, the woman was confronted with a sense of meaninglessness due to her inability to have children: “I was very positive, but when I thought about not being able to, I was overcome by a sense of meaninglessness.” However, Gertrūda points out that “life itself is the meaning; living life has a much greater meaning than waiting for something and not getting it.” The woman also realises that it is her relationship with her husband that is most important: “and so a child is no longer my goal, but a quality relationship”, “the whole therapy process for me revolved around the relationship, it seems, from that anger towards my husband to a realisation, like in * *, that the relationship with him is what matters most”, “I’ve gathered all the drawings and I see that, based on this, the relationship is the foundation; when I call my husband a fool, the children repeat ‘fool, fool’ after me, and then everyone is unhappy.” She realises that nurturing a relationship requires conscious effort: “we really need to show more love for one another”, “unity, harmony, balance, respect—that is the foundation, just as this tree is so strong and harmonious”, “I want to grow together and move towards complete acceptance.”

**2. Letting go of the baby.** From day one, the participant joined the therapy group with the aim of letting go of the baby: “I’d do it in a heartbeat,” but she realised that it would take time and effort: “Well, there’s no need to rush into anything here or try to act all clever.” Nevertheless, over time, the woman stated that she had managed to let go; she identified the turning point as writing a letter to her unborn child: “When I wrote that letter, I poured so much out; it felt as though I had let go.” The research participant came to the therapy group having already given her unborn baby a name, which she had chosen whilst meditating. This helped Gertrūda to come to terms with her loss more deeply. The woman kept drawing and talking about symbols: “Here is a coffin, like a way of giving meaning, a farewell to my Gabrielė,” “The funeral here is just like that.” This also suggests that Gertrūda managed to come to terms with, acknowledge and accept the loss. This assumption is also confirmed by her account of how, after many years of silence, she told her child about the loss of her first child: “I said there was a little baby in my tummy, and then it died. He took it all very calmly.” Gertrūda modelled her unborn child in her final therapy session, in a piece representing her family: “I wanted her to be part of our family portrait too.” Furthermore, during the sessions, the woman spoke of the changed feelings associated with the miscarriage: “I can talk about it completely calmly; I even posted about it on Facebook.” This change is also reflected in the symbols in her drawings, which she describes as follows: “such a transformation—I drew how that pain turns into an angel”, “the little heart was like it was bleeding, but now it’s healed, bright.” Finally, the woman even compared her relationship with her unborn child before therapy: “because when I came back from the hospital, that little one remained in my heart, but I put him in prison” and after it: “all that pain, those reproaches, the story of that little one, how I lost him there—I’ve definitely let go of some of it.”

**3. Changes in relationships with loved ones**. The woman notices a change in her relationship with her husband. In the first part of the therapeutic process, Gertrūda spoke of the anger she felt towards her husband because of his inappropriate behaviour; she realised that deep down she blamed him for the loss: “And I felt such deep sadness, anger, that he behaved like that towards a pregnant woman,” “How could he do that?”, “I’ve got so much of that anger built up and it just drags on and on.” However, later the woman stated: “I used to expect so much from my husband; many things used to hurt me, but now very little,” “I used to shout and get angry with him, but now I feel as though a sense of acceptance has emerged.” Gertrūda also mentioned changes in her relationships with other people: “I used to lecture them; I didn’t like my own parents or my husband’s, but now it’s really different—they’re above me and I accept them unconditionally, full stop. No criticism,” “that friend really used to get on my nerves, but now I understand—there’s no point getting involved in that relationship if it’s toxic, if it’s harmful.” The participant realises that it is important to set boundaries in any relationship: “and you have to set boundaries so they don’t walk all over you.” Gertrūda has started setting boundaries with her children too: “Before, I used to give them whatever they wanted—if they wanted this, I’d give it; if they wanted that, I’d give it all away. I couldn’t say no, but now I say—no, and so what? Nobody’s going to die, they understand.”

**4. The theme. Efforts to create a harmonious relationship with oneself.** Constant work on herself, self-development, has become very important to Gertrūda: “I do my practices”, “it’s important to me that I grow within myself; that is my diamond.” The importance of self-reflection also comes to the fore: “I keep a gratitude journal”, “I’m writing a journal like that now, well, I’ll publish it later”. The woman explains that she has discovered that it is precisely taking care of herself that helps her maintain harmonious relationships and look after others: “I used to jump at what others wanted and do it, and then I’d get scattered and walk around angry”, “Because of a lack of self-confidence, I wanted so much of everything, but now mmmm [pause] I’ve moved into a connection with myself, knowing what I want, and when I listen to myself, then I can accept others unconditionally and trust the universe itself, not people, and that helps when it’s hard to accept others.” The participant knows what kind of life she wants: “I want to feel harmony myself, to trust myself, to trust the universe—to accept, to trust [pause, ponders], well, to control less and trust more.” Nevertheless, the woman admits that she still lacks self-confidence: “I’m starting to love myself, but when it comes to taking action, taking the initiative, I still fall short”, “I feel as though I’m all tangled up inside—I know my path, but when it comes to just getting on with it, I can’t.” Summarising the data from the thematic analysis, several of the most striking experiences of the study participant Gertrūda during the art therapy process can be identified; they are presented below (see Table 7).

In summary, it can be said that Gertrude’s toxic relationship with herself has become apparent, manifesting as constant dissatisfaction with her current situation, blaming herself and others, an excessive pursuit of perfection, and a need for control. For this reason, she does not allow herself to feel strong emotions, suppressing, ignoring and denying them, and isolating herself from those around her. However, having acknowledged her repressed pain, and having processed and identified her suppressed emotions (helplessness, anger, fear, sadness, guilt, emptiness, a trance-like state, a sense of objectification, etc.) through the art therapy process, she found the strength to share her experience of loss with others on social media and through interactions with children. Giving the baby a name and planting a plant helped the woman to make sense of her loss, acknowledge it, find a place for it in her life, and let it go. This is confirmed by the woman’s account of her desire to draw her unborn child in a family portrait. Another important aspect that emerged during the therapeutic process and in the thematic analysis data was the heightened importance of relationships with oneself and others. The research participant began to devote considerable attention to working on herself, mindfulness practices, nurturing her relationship with her husband, accepting others, and setting clear boundaries.

## 5. Discussion

**Figure 1:**
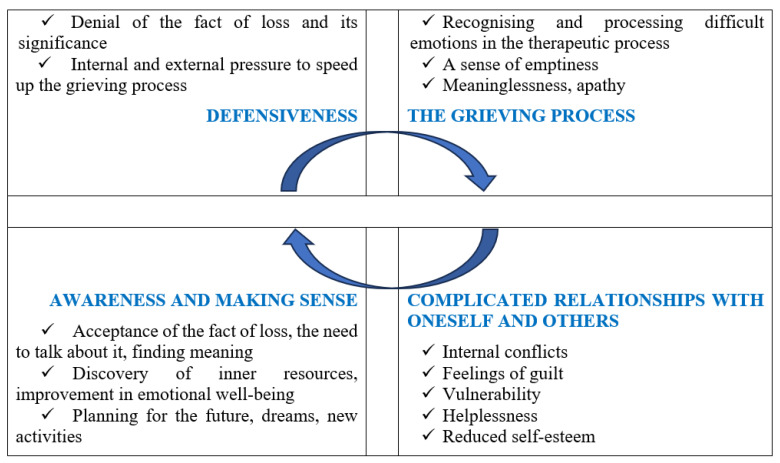
Themes revealed in the thematic analysis of all study participants (compiled by the author).

Summarising the thematic analysis data, four main themes emerged from the reflections of all study participants: defensiveness, the grieving process, a complicated relationship with oneself and others, and the realisation and making sense of loss. The systematised data are presented in Figure 1.

The thematic analysis data for all study participants reveal defensiveness, manifested in previously suppressed or downplayed feelings related to loss, which the women recognised and worked through during the therapeutic process, or in internal and external pressure to hasten the grieving process and return to ‘normal’ life more quickly. Two women, who had experienced a miscarriage more than 10 years ago, recognised their own defensiveness—the denial of the significance and importance of the loss, the suppression of difficult emotions, not allowing themselves to feel them, and efforts not to think about the loss. A woman who lost her baby three months ago experienced defensiveness by feeling external (from those around her) and internal pressure to ‘pull herself together’ more quickly, to stop grieving and to stop crying. Conversely, during the art therapy process, the study participants allowed themselves to grieve by acknowledging and processing difficult emotions. The women’s accounts revealed the experience of difficult emotions such as anger, sadness, a sense of meaninglessness, helplessness, apathy, a feeling of emptiness, and a sense that their lives had fallen apart. A complicated relationship with themselves and others also emerged. The study participants faced reduced self-esteem, self-blame, internal conflicts, increased sensitivity, vulnerability, rejection by those around them, and a need to distance themselves from certain people. On the other hand, when analysing the dynamics of the therapeutic process, it is notable that the women became aware of their loss and gave it meaning. Through this, the study participants grew stronger, discovered inner resources, and began to form visions of the future and dreams.

The results of this study can be applied in practice to develop recommendations for medical staff regarding pregnancy terminations, thereby helping to improve hospital care. The study’s findings may broaden understanding of the services available in hospitals, clinics and other facilities where women learn of the loss of their baby, with a view to alleviating their grief. Research indicates that women who have experienced a spontaneous pregnancy loss face symptoms of depression, post-traumatic stress disorder, distress, prolonged and complicated grief, and experience anxiety, fear, and sleep and eating disorders (Farren et al., 2020; Khodakarami et al., 2017; Kulathilaka et al., 2016; Lok et al., 2010; Seftel, 2020; Herbert et al., 2022; Aventin et al., 2025; Shetty et al., 2025). Most of these symptoms were also observed in the participants’ reflections. Although the aim of our study was not to measure depression, it can nevertheless be assumed that the study participants experienced symptoms of complicated grief. The thematic analysis of the women’s responses revealed aspects of apathy, emptiness, indifference and a loss of joy in life. Another long-term study showed that miscarriage can cause significant emotional distress, and for some women, psychological symptoms may persist for several years following the loss of pregnancy (Broen et al., 2005). A similar aspect emerged from the thematic analysis of our study participants’ data—the theme of defensiveness. The women found that not allowing themselves to grieve, suppressing their emotions, and downplaying them did not provide the relief they so desired; this later manifested as negative consequences in their subsequent lives. The suppressed emotions did not disappear—the women cried and grieved, despite the fact that 10 or 14 years had passed. In other studies, pregnancy loss is associated with an increased risk of frequent mental health disorders in women later in life (Shen et al., 2024). The reflections of several women highlight a lack of understanding from others, including doctors, as well as the downplaying, trivialisation and indifference towards the difficult feelings they experienced; this is also reflected in other similar studies (Jacob et al., 2019; Cuenca, 2023).

Although our study is qualitative and therefore does not aim to determine the impact of art therapy, the thematic analysis data nevertheless reveal experiences of accepting, coming to terms with and making sense of loss. This is reflected in Gertrūda’s theme ‘Making sense of loss’, Jolita’s theme ‘The process of coming to terms with loss’, and Elzė’s themes ‘Acceptance of loss’ and ‘Wanting to start living life’. The importance of making sense of loss is revealed in the thematic analysis of all study participants. Through the art therapy process, the women may have established a connection with their lost child, creatively expressing to them what they could not say, conveying their love and wisdom, recognising and processing difficult emotions, exploring their relationships with themselves and others, and allowing themselves to envision the future. Therefore, these experiences are linked to the work of researchers (Zahmatkesh et al., 2024), whose study found an improvement in psychological well-being following art therapy sessions. Andrus’s (2020) study examined the experiences of seven women who exhibited artworks about their losses or participated in the exhibition as witnesses. The results show that reflecting on the artworks helped with reintegration during the trauma healing process and that participating in a supportive community exhibition had therapeutic benefits. Before the exhibition, the women felt isolated; during the exhibition, they felt recognised; and after the exhibition, they understood the significance of such an exhibition. Participation reduced stigma and brought visibility to the rarely discussed topic of pregnancy loss (Andrus, 2020). The importance of the therapeutic group emerged in the thematic analysis of our study participants.

We also wish to present the ideas that have emerged for exploring new areas of research and for the practical application of the findings. This study has undoubtedly opened up new areas of research, provided insights for future studies, and suggested practical ways to apply the findings. Although studies have been conducted abroad to date, revealing the emotional state of women who have experienced pregnancy loss, it would be possible to compare how this changes depending on whether the woman in question is a mother who has already had children or a young, childless woman who has not yet experienced pregnancy loss. We believe that such a study could be carried out in the future.

Furthermore, it is important to develop quantitative studies to help determine the impact of art therapy on women who have experienced such a loss, as there is still a lack of evidence confirming the effectiveness of these methods. By employing other qualitative analysis methods (e.g., grounded theory), it would be possible to validate certain assumptions or develop theories. It would also be possible to explore other aspects of women’s experiences and compare them with the experiences of women who have undergone induced abortion during the art therapy process. It would be interesting to investigate not only the experiences of mothers but also those of men whose partners have experienced stillbirth. Another area of research could be the experiences of women who have experienced stillbirth and the impact of interventions, as there were approximately 2 million stillbirths worldwide in 2019 (Hug et al., 2021). This is a deeply traumatic and complex experience, leaving mothers and parents with intense grief (Shamai & Metzl, 2025).

## 6. Conclusions

An inductive thematic analysis of the research data revealed four themes characterising the core experiences of women who have experienced spontaneous pregnancy loss, as expressed by the study participants following art therapy sessions. These are: (1) Defensiveness, manifested in the denial of the fact of loss and the pressure from those around them to speed up the grieving process. (2) The experience of the grieving process, most often characterised by recognisable difficult emotions expressed by the women when discussing their drawings, a sense of emptiness, meaninglessness and apathy. (3) A complicated relationship with oneself and others—this involves internal conflicts, feelings of guilt, vulnerability, helplessness and reduced self-esteem. (4) Awareness and finding meaning—this involves acknowledging the reality of loss, the need to talk about it, discovering inner resources, an improvement in emotional well-being, planning for the future, articulating dreams, and planning new activities. After the art therapy sessions, all the women who took part in the study felt better, discovered ways to devote attention and time to themselves, and began to make plans for the future, which rekindled a sense of meaning in their lives.

**Limitations and shortcomings of the study.** We encountered several difficulties during the study. Initially, it was planned to conduct all art therapy sessions in the same location, where the participants could find the same art materials each time. However, due to the announcement of the first nationwide lockdown, the therapeutic process was interrupted for two weeks and subsequently continued remotely. For this reason, the women used the materials they had available, which may have influenced the formal elements of the drawings. Furthermore, the participants lost the opportunity to be physically close to one another, which is very important in group therapy, as togetherness is one of the most important aspects of such therapy.

## Figures and Tables

**Table 1:** Themes, methods, objectives and tools of the art therapy sessions (compiled by the author).

Theme of the Art Therapy Session	Objectives, Methods and Tools of Art Therapy
Life recently	Introduction, getting started with the art therapy process. All materials (acrylics, gouache, oil paints, wax crayons, pencils, felt-tip pens). Creating a piece of art that reflects life recently.
2. Emotions and feelings related to the loss of a child	Recognising and processing difficult emotions that may not have been fully acknowledged. Paints. Naming emotions using the wheel of emotions. Processing emotions using paint.
3. The heaviest and most intense feeling accompanying the experience of loss	Identifying and processing the most difficult feeling. Acrylic. Painting the strongest feeling.
4. Letting go—saying goodbye	Saying goodbye to a lost child. All methods. Write or draw a letter, a story or a poem with illustrations for the child. Plant a seed in a pot with soil; once it has sprouted, transplant the flower to a beautiful spot.
5. Relationship with one’s body	Explore your relationship with your body. All resources. Create a life-size silhouette that reflects your relationship with your body.
6. Relationship map	Explore relationships and how they change; discover what satisfies you in this area and what you would like to change. All methods. Create a map reflecting relationships with those around you.
7. Self-esteem	Explore self-assessment. All methods. Create a piece of work representing self-esteem.
8. Internal resources	Discover internal resources. All methods. Create a piece of work that reveals your inner resources.
9. Wishes and dreams	Allow yourself to dream, to wish, and to take steps towards realising your dreams. All means. Create a piece of work representing desires and dreams.
10. Summary of the therapeutic process	Summarise the therapeutic process, identify the most important points. All methods. Create a piece of art that reflects the summary of the therapeutic process.

**Table 2:** Main themes and sub-themes of the results of Elzė’s thematic analysis (compiled by the author).

Theme	Sub-Themes
1. A difficult experience of hospitalisation	1.1. Helplessness, fear and guilt 1.2. Indifference of doctors 1.3. Physical and emotional pain: ‘My body is about to break’ 1.4. Loneliness and efforts to protect her husband 1.5. The painful attitude of other patients towards life
2. “That knocked me for six”	2.1. The feeling that life has fallen apart 2.2. The collapse of her greatest dream in life: to have a daughter 2.3. Hiding her pain: “My smile is fake, my cheeks are sunken” 2.4. Symptoms of psychological trauma 2.5. Physical tension 2.6. Reduced need for social interaction, withdrawal from loved ones
3. Coming to terms with loss	3.1. Expressing grief through the need to talk about the loss 3.2. A sense of peace found whilst visiting her daughter’s grave 3.3. A turning point through the interpretation of significant symbols 3.4. Letting go and accepting one’s situation 3.5. The importance and influence of the art therapy group
4. “I want to start living my life”	4.1. Efforts to create a dream job 4.2. Practising new activities 4.3. The fear of having another baby has disappeared

**Table 3:** Experiences revealed in Elzė’s thematic analysis (compiled by the author).

A difficult experience of hospitalisation	“It knocked me for six”
> Experiences of helplessness, fear and guilt. > Unbearable physical and emotional pain, when it feels like you cannot go on. > Experiencing loneliness, pain when confronted with a different perspective on life.	> A sense that your life is falling apart. > The collapse of your greatest dreams. > Internal and external pressure to feel positive emotions more quickly. > Sleep disturbances, mood swings, persistent and recurring experiences related to the loss; reliving the experience in memories, dreams or nightmares. > Physical tension, trying to help oneself by following bodily impulses, talking about it. > Encountering avoidance from friends, a natural decrease in the need for social interaction.
Coming to terms with the loss	“I want to start living my life”
> Grieving, accepting difficult emotions, and talking about them helps to ease the pain. > A sense of peace found whilst visiting her daughter’s grave. > Finding meaning in the loss through certain symbols.	> Coming to terms with one’s situation, letting go, and finding support in faith in God. > A new desire to try new activities, to take on a new, dream job. > The fear of planning another pregnancy has disappeared.

**Table 4:** Main themes and sub-themes of the results of Jolita’s thematic analysis (compiled by the author).

Theme	Sub-Themes
1. The uncontrollable grieving process	1.1. Self-blame 1.2. Withdrawal and isolation 1.3. Denial of the fact of loss 1.4. Bubbling emotions 1.5. Tension in the body
2. ‘Life simply vanished’	2.1. Failed expectations 2.2. The disconnect between desires and current life 2.3. The indifference of those around me: “Am I adopted after all?” 2.4. Unresolved issues complicating relationships 2.5. A terrifying emptiness
3. “Well then? I have no self-worth”	3.1. Why don’t they love me—“I’m as useless as a whistle on a windy day” 3.2. Unstable self-esteem 3.3. Distancing oneself from people who have children
4. The process of coming to terms with loss	4.1. Coming to terms with and making sense of the loss by giving it a name 4.2. Discovering the positive aspects—“And I realise that I cannot change this situation, and through this I am becoming a stronger person.” 4.3. The pain leaves me, remaining in the pages 4.4. The importance of the therapy group

**Table 5:** Experiences revealed in Jolita’s thematic analysis (compiled by the author).

An uncontrollable grieving process	Life simply ceased to exist
> The release of suppressed, unexpressed feelings and emotions during the art therapy process: self-blame, anger, rage, fear, jealousy, sadness, physical tension, feelings of betrayal and loneliness. > A desire to distance oneself from others, their indifference. > Suppression of the reality of loss and difficult emotions.	> Failed life plans, shattered dreams, a sense of meaninglessness. > A disconnect between the life one wants and the life one has. > Experiencing a frightening sense of emptiness, indifference, and numbness. > Complicated relationships with those around you.
“Well, there’s no self-worth”	The process of coming to terms with loss.
> The feeling of being unloved and misunderstood. > Fear of being oneself, constant efforts to appear better than one is. > A desire to distance oneself from those who have children. > Helplessness, fear, emptiness, worthlessness.	> Coming to terms with the loss and finding meaning by giving a name to the unborn child. > Recognising the positive aspects of one’s current situation. > Emotionally experiencing the release of pain through watercolour painting. > The importance of the therapeutic group in the process of coming to terms with the loss.

**Table 6:** Main themes and sub-themes of the results of Gertrūda’s thematic analysis (compiled by the author).

Theme	Sub-Themes
1. The spectrum of difficult emotions in a relationship	1.1. Centrifuge 1.2. Not allowing oneself to grieve, the desire to be strong 1.3. A toxic relationship with oneself as a meat grinder: ‘you get stuck and spin round’
2. Making sense of loss	2.1. The importance of relationships and the meaning of life discovered 2.2. Letting go of a child 2.3. Changes in relationships with loved ones 2.4. Efforts to build a harmonious relationship with oneself

**Table 7:** Experiences revealed in Gertrūda’s thematic analysis (compiled by the author).

Spectrum of difficult emotions	Making sense of loss
> Low self-esteem, self-blame, perfectionism; > Defensiveness; > Not allowing oneself to grieve, suppression of difficult emotions, denial; > Helplessness, fear, emptiness, worthlessness.	> Coming to terms with the loss, a growing desire to talk to those around you about the loss; > The importance of relationships with those around us, particularly with one’s partner; setting boundaries; and the newly acquired ability to accept others’ imperfections; > Conscious work on oneself, with the aim of self-acceptance; > The discovery of the meaning of life.

## Data Availability

The original contributions presented in this study are included in the article. Further inquiries can be directed to the corresponding author.

## References

[B1-behavsci-16-00801] Andrus M. (2020). Exhibition and film about miscarriage, infertility, and stillbirth: Art therapy implications. Art Therapy.

[B2-behavsci-16-00801] Arnold R. (2023). Grieving artists: Influences of loss and bereavement on visual art making. The Arts in Psychotherapy.

[B3-behavsci-16-00801] Aventin Á., Robinson M., White J., Galeotti M. (2025). Recurrent pregnancy loss, psychological distress and wellbeing support for women: A mixed-methods analysis. BMC Women’s Health.

[B4-behavsci-16-00801] Avis K. A., Stroebe M., Schut H. (2021). Stages of grief portrayed on the internet: A systematic analysis and critical appraisal. Frontiers in Psychology.

[B5-behavsci-16-00801] Bat Or M., Garti D. (2018). Art therapist’s perceptions of the role of the art medium in the treatment of bereaved clients in art therapy. Death Studies.

[B6-behavsci-16-00801] Bilardia J. E., Sharp G., Payne S., Temple-Smith M. J. (2021). The need for improved emotional support: A pilot online survey of Australian women’s access to healthcare services and support at the time of miscarriage. Women and Birth: Journal of the Australian College of Midwives.

[B7-behavsci-16-00801] Blomdahl C., Wijk H., Guregård S., Rusner M. (2018). Meeting oneself in inner dialogue: Manual-based Phenomenological Art Therapy as experienced by patients diagnosed with moderate to severe depression, a qualitative study. The Arts in Psychotherapy.

[B8-behavsci-16-00801] Braun V., Clarke V. (2006). Using thematic analysis in psychology. Qualitative Research in Psychology.

[B9-behavsci-16-00801] Braun V., Clarke V. (2013). Successful qualitative research: A practical guide for beginners.

[B10-behavsci-16-00801] Bright K. S., Charrois E. M., Mughal M. K., Wajid A., Mcneil D. A., Stuart S., Hayden K. A., Kingston D. (2020). Interpersonal psychotherapy to reduce psychological distress in perinatal women: A systematic review. International Journal of Environmental Research and Public Health.

[B11-behavsci-16-00801] Broen A. N., Moum T., Bødtker A. S., Ekeberg O. (2005). The course of mental health after miscarriage and induced abortion: A longitudinal, five-year follow-up study. BMC Medicine.

[B12-behavsci-16-00801] Callen T. (2022). Narratives of grief: The experience and development of grief for young adults who have been parentally bereaved in adolescence. Doctoral thesis.

[B13-behavsci-16-00801] Chang C.-C., Wang Y.-H. (2021). Using phenomenological methodology with thematic analysis to examine and reflect on commonalities of instructors’ experiences in MOOCs. Education Sciences.

[B14-behavsci-16-00801] Coenen C. (2018). Shattered by grief: Picking up the pieces to become whole again.

[B15-behavsci-16-00801] Cuenca D. (2023). Pregnancy loss: Consequences for mental health. Frontiers in Global Women’s Health.

[B16-behavsci-16-00801] Farren J., Jalmbrant M., Falconieri N., Mitchell-Jones N., Bobdiwala S., Al-Memar M., Tapp S., Van Calster B., Wynants L., Timmerman D., Bourne T. (2020). Posttraumatic stress, anxiety and depression following miscarriage and ectopic pregnancy: A multicenter, prospective, cohort study. American Journal of Obstetrics and Gynecology.

[B17-behavsci-16-00801] Gaižauskaitė J. (2019). Moterų emocinė būklė po persileidimo *[Women’s emotional state after a miscarriage]*. Master’s thesis.

[B18-behavsci-16-00801] Geniušas S. (2021). Kas yra skausmas? *[What is pain?]*.

[B19-behavsci-16-00801] Giorgi A., Giorgi B., Morley J., Willig C., Stainton Rogers W. (2017). The descriptive phenomenological psychological method. The Sage handbook of qualitative research in psychology.

[B20-behavsci-16-00801] Giorgi A. P., Giorgi B. M., Camic P. M., Rhodes J. E., Yardley L. (2003). The descriptive phenomenological psychological method. Qualitative research in psychology: Expanding perspectives in methodology and design.

[B21-behavsci-16-00801] Herbert D., Young K., Pietrusińska M., MacBeth A. (2022). The mental health impact of perinatal loss: A systematic review and meta-analysis. Journal of Affective Disorders.

[B22-behavsci-16-00801] Hogan S. (2020). Arts therapies and gender issues: International perspectives on research.

[B23-behavsci-16-00801] Hug L., You D., Blencowe H., Mishra A., Wang Z., Fix M. J., Wakefield J., Moran A. C., Gaigbe-Togbe V., Suzuki E., Liu Y. (2021). A neglected tragedy: The global burden of stillbirths. The Lancet.

[B24-behavsci-16-00801] Jacob L., Gerhard C., Kostev K., Kalder M. (2019). Association between induced abortion, spontaneous abortion, and infertility respectively and the risk of psychiatric disorders in 57,770 women followed in gynecological practices in Germany. Journal of Affective Disorders.

[B25-behavsci-16-00801] Khodakarami B., Mafakheri B., Shobeiri F., Soltanian A., Mohagheghi H. (2017). The effect of Fordyce Happiness cognitive behavioral counseling on the anxiety and depression of women with spontaneous abortion. Journal of Pharmaceutical Sciences and Research (JPSR).

[B26-behavsci-16-00801] Kouriatis K., Brown D. (2013). Therapists’ experience of loss: An interpretative phenomenological analysis. Omega.

[B27-behavsci-16-00801] Kulathilaka S., Hanwella R., de Silva V. A. (2016). Depressive disorder and grief following spontaneous abortion. BMC Psychiatry.

[B28-behavsci-16-00801] Kübler-Ross E., Wessler S., Avioli L. V. (1972). On death and dying. JAMA.

[B29-behavsci-16-00801] Kvale S. (1983). The qualitative research interview: A phenomenological and a hermeneutical mode of understanding. Journal of Phenomenological Psychology.

[B30-behavsci-16-00801] Lok I. H., Yip A. S., Lee D. T., Sahota D., Chung T. K. (2010). A 1-year longitudinal study of psychological morbidity after miscarriage. Fertil Steril.

[B31-behavsci-16-00801] Malchiodi C. A. (2020). Trauma and expressive arts therapy: Brain, body, and imagination in the healing process.

[B32-behavsci-16-00801] Malterud K., Siersma V. D., Guassora A. D. (2016). Sample size in qualitative interview studies: Guided by information power. Qualitative Health Research.

[B33-behavsci-16-00801] Markin R. D., Zilcha-Mano S. (2018). Cultural processes in psychotherapy for perinatal loss: Breaking the cultural taboo against perinatal grief. Psychotherapy.

[B34-behavsci-16-00801] Matulaitė A. (2013). Kai „tavo kūnas tiesiog išprotėja“: Įkūnytas nėštumo patyrimas *[When “your body just goes crazy”: The embodied experience of pregnancy]*. Doctoral dissertation.

[B35-behavsci-16-00801] McKernan A. (2020). Healing miscarriage trauma through expressive arts therapy, ritual, and healing circles: A critical literature review. Master’s thesis.

[B36-behavsci-16-00801] Mickūnas A., Jonkus D. (2014). Fenomenologinė filosofija ir jos šešėlis *[Phenomenological philosophy and its shadow]*.

[B37-behavsci-16-00801] Ministry of Health of the Republic of Lithuania (2018). Dėl meno terapijos paslaugų teikimo reikalavimų patvirtinimo *[Regarding the approval of requirements for the provision of art therapy services]*.

[B38-behavsci-16-00801] Moran D. (2000). Introduction to phenomenology.

[B39-behavsci-16-00801] Neimeyer R. A., Baldwin S. A., Gillies J. (2006). Continuing bonds and reconstructing meaning: Mitigating complications in bereavement. Death Studies.

[B40-behavsci-16-00801] Seftel L. (2020). Art therapy and pregnancy loss: A secret grief. Therapeutic arts in pregnancy, birth and new parenthood.

[B41-behavsci-16-00801] Sezer K. S., Aki E. (2024). “*It is as if I gave a gift to myself*”: A qualitative phenomenological study on working adults’ leisure meaning, experiences, and participation. Behavioral Sciences.

[B42-behavsci-16-00801] Shamai M. G., Metzl E. (2025). Your life within me: Exploring a visual art journal group intervention for women who experienced stillbirth. The Arts in Psychotherapy.

[B43-behavsci-16-00801] Shen Q., Zhong W., Wang X., Fu Q., Mao C. (2024). Associations between pregnancy loss and common mental disorders in women: A large prospective cohort study. Frontiers in Psychiatry.

[B44-behavsci-16-00801] Shetty A., Issac A., Dhiraaj S., Vr V., Thimappa L., Balakrishnan D., Nath B., Sinha S., Singh S., Mishra P., Halemani K. (2025). Global prevalence of post-miscarriage anxiety, depression, and stress: A systematic review and meta-analysis. Journal of Global Health.

[B45-behavsci-16-00801] Tavoli Z., Mohammadi M., Tavoli A., Moini A., Effatpanah M., Khedmat L., Montazeri A. (2018). Quality of life and psychological distress in women with recurrent miscarriage: A comparative study. Health Qual Life Outcomes.

[B46-behavsci-16-00801] Taylor H. S., Pal L., Seli E. (2020). Recurrent early pregnancy loss. Speroff’s clinical gynecologic endocrinology and infertility.

[B47-behavsci-16-00801] Tekutienė M. (2022). Savaiminį nėštumo nutrūkimą išgyvenusių moterų patyrimai dailės terapijos procese *[The experiences of women who have experienced a miscarriage in the art therapy process]*. Master’s thesis.

[B48-behavsci-16-00801] Wahlbeck H., Kvist L. J., Landgren K. (2020). Art therapy and counseling for fear of childbirth: A randomized controlled trial. Art Therapy.

[B49-behavsci-16-00801] Zahmatkesh M., Faal Siahkal S., Alahverdi F., Tahmasebi G., Ebrahimi E. (2024). The role of art therapy on quality of life of women with recent pregnancy loss: A randomized clinical trial. PLoS ONE.

[B50-behavsci-16-00801] Žydžiūnaitė V., Sabaliauskas S. (2017). Kokybiniai tyrimai: Principai ir metodai *[Qualitative research: Principles and methods]*.

